# Critical Care Nurses’ Practices in Clinical Alarm Management: Barriers and Predictors From a Mixed‐Methods Study in the Southern West Bank, Palestine

**DOI:** 10.1155/nrp/4564347

**Published:** 2026-01-11

**Authors:** Fuad Farajalla, Mousa Farajallah, Nesreen Alqaissi, Mohammad Qtait

**Affiliations:** ^1^ Nursing College, Palestine Polytechnic University, Hebron, State of Palestine, ppu.edu; ^2^ Palestine Polytechnic University, Hebron, State of Palestine, ppu.edu

**Keywords:** alarm fatigue, alarm management, barriers, critical care nursing, Palestine, patient safety, predictors

## Abstract

**Background:**

Clinical alarms serve as vital safety mechanisms in Critical Care Units (CCUs); however, alarm fatigue and ineffective management remain global safety concerns. Evidence from Palestine is scarce, where staffing shortages and limited resources may further affect alarm management practices.

**Aim:**

This study aimed to assess critical care nurses’ practices regarding clinical alarm management, identify perceived barriers, and examine predictors influencing these practices in the southern West Bank, Palestine.

**Methods:**

A cross‐sectional design was used, involving 146 critical care nurses who completed a structured questionnaire assessing alarm management practices and barriers, with two open‐ended questions. Quantitative data were analyzed using SPSS for descriptive and inferential statistics, while qualitative responses were thematically analyzed.

**Results:**

The mean alarm‐practice score was 3.56 out of 5. The majority of participating nurses had poor alarm management practices (65.8%). Female (*β* = 0.31, *p* < 0.001), alarm training (*β* = 0.22, *p* = 0.004), and younger age (*β* = −0.21, *p* = 0.025) significantly predicted better practices. Major barriers were inadequate staffing and frequent false alarms. Four themes emerged: alarm fatigue, workload issues, limited training/support, and environmental or technical obstacles.

**Conclusion:**

Alarm management practices among critical care nurses were below the expected standard. Addressing staffing limitations, strengthening training programs, and implementing supportive policies are essential to enhance alarm safety in Palestinian CCUs.

## 1. Introduction

Clinical alarms are essential safety mechanisms in modern healthcare, and they are intended for the purpose of alarming clinicians about significant changes in the conditions of patients by way of audible and visual alerts provided by devices like cardiac monitors, ventilators, and infusion pumps [1]. The alarms assume a critical role in the provision of timely intervention in critical care units (CCUs), primarily because the patient might not express changes in their condition [2]. The efficacy of the alarm rests not only in the technology but also in the nurses providing the monitoring service [3]. Critical care nurses’ (CCNs) skills in prioritization of the alarm and the way they respond to the alarm are therefore important in patient safety in high‐acuity units [4].

In spite of their critical role, alarm management practices are affected by numerous human, technical, and organizational factors, including high alarm rates, heavy workload, understaffing, and inadequate training on alarm systems [4, 5]. These operational barriers are distinct from alarm fatigue, a psychological and behavioral phenomenon that develops after repeated exposure to non‐actionable or false alarms, leading to reduced attentiveness and inappropriate responses to alarm sounds [6, 7]. While alarm fatigue reflects an individual‐level response, issues such as understaffing, lack of professional expertise, and alarm complexity primarily reflect system and educational deficiencies.

The Joint Commission has consistently identified alarm‐related issues among the top hazards in healthcare technology and included alarm safety in its National Patient Safety Goals [8, 9]. Global studies emphasize the universality of the alarm management problem. American, South Korean, and Taiwanese studies verify the prevalence of alarm fatigue and how repeated false alarms diminish the effectiveness of staff and create opportunities for critical events [7, 8, 10]. A study conducted in Kenya revealed that most CCNs (81.6%) required training in clinical alarm practices [4]. Specific staff training programs have been effective in enhancing nurse awareness and understanding of alarm systems, but the durability of such initiatives in alarm stress reduction is questionable [11].

Regionally, in the Middle East, Iranian, Egyptian, and Lebanese evidence indicates alarm fatigue and questionable practices. Nurses in Iranian studies pinpointed the high frequency of false alarms and lack of training as major concerns [12]. In an Egyptian study, findings revealed over three‐quarters of the nurses had unsatisfactory practice regarding clinical alarms [13], and Lebanon‐based nurses indicated nuisance alarms and staffing as major challenges [14]. These regional studies point to the necessity for contextually and culturally specific solutions in the improvement of alarm safety.

In the West Bank, the evidence related to the management of alarms has just begun to emerge. Findings from a recent investigation in the region of the Northern and Central West Bank revealed that over 60% experience alarm fatigue, closely related to increased levels of stress [3]. However, no study in Palestine has comprehensively examined alarm‐management practices or identified their predictors, and none in the southern West Bank has used a mixed‐methods design to investigate both aspects simultaneously. This gap limits understanding of how behavioral, organizational, and contextual factors shape nurses’ responses to clinical alarms. The current research aims to close this research gap by adopting the mixed‐methods strategy. This gap limits understanding of how behavioral, organizational, and contextual factors shape nurses’ responses to clinical alarms. Beyond operational barriers, broader systemic pressures also shape nursing performance; for example, Aqtam et al. [15] demonstrated how ongoing political stressors undermine resilience among nursing students, reflecting the demanding environment in which healthcare providers work. Such contextual constraints may intensify susceptibility to alarm fatigue and compound resource‐related barriers, further highlighting the need for this investigation.

Therefore, this study aims to investigate CCNs’ practices regarding clinical alarm management in the southern West Bank. Specifically, it (a) assesses nurses’ alarm‐management practices; (b) identifies the perceived barriers that hinder timely and effective responses to clinical alarms; and (c) examines the demographic and professional predictors influencing these practices using a mixed‐methods approach.

## 2. Methods

### 2.1. Design and Setting

This study adopted a cross‐sectional descriptive‐correlational design, with embedded qualitative elements, using a convergent approach in which qualitative responses were analyzed alongside quantitative results to provide contextual depth and triangulation of findings. Qualitative insights helped interpret statistical trends, particularly regarding barriers and predictors of alarm management practice. The study was conducted in the CCUs of three governmental hospitals in the southern West Bank, Palestine: Beit Jala governmental hospital, Hebron governmental hospital, and Dura governmental hospital, representing the largest public healthcare facilities in the region. These sites were selected purposively to reflect diverse patient volumes, staffing levels, and available technological resources.

### 2.2. Population and Sampling

The target population was nurses working in CCUs at governmental hospitals in the southern West Bank, Palestine. Data on the number of CCNs were gathered by directly contacting the nursing directors of the hospitals, as no published reports were available, and a population of 200 CCNs was estimated. Raosoft online calculated a minimum sample of 132 (95% confidence level, 5% margin of error, 50% response distribution).

Eligible nurses included those currently employed in CCUs who were available for data collection and could provide consent. Nurse managers and nurses with less than 1 year of experience were excluded to limit potential biases related to limited patient contact and limited responsibility for clinical alarm management. Participants were recruited using convenience sampling.

### 2.3. Instrumentation

#### 2.3.1. Quantitative Instrument

It consisted of a questionnaire divided into three sections. The first collected socio‐demographic data (Table 1), developed based on prior studies [3, 4]. The second assessed alarm management practices using a 9‐item tool originally developed by Meng’anyi et al. [4]. Items 4 and 5 were modified for specificity with author approval.

**Table 1:** Distribution of demographic information among participants (*N* = 146).

Demographics	Categories	Frequency	Percentage
Gender	Male	69	47.3
	Female	77	52.7

Marital status	Single	52	35.6
	Married	89	61.0
	Other	5	3.4

Level of education	Diploma	29	19.9
	Bachelor’s degree	92	63.0
	Master’s degree or higher	25	17.1

Experience	1–5 years	60	41.1
	6–10 years	45	30.8
	> 10 years	41	28.1

Alarm training	Yes	70	47.9
	No	76	52.1

Each item was rated on a 5‐Point Likert‐Type Scale (1 = *never* to 5 = *always*). Following the developers’ guidance, item scores were averaged to generate a composite practice score, with higher scores indicating better alarm management. Although Meng’anyi et al. [4] did not specify a numerical cutoff for classifying alarm‐management practice, they described alarm performance as ideally approaching very high levels. To operationalize practice categories in a reproducible manner, we applied the Bloom‐based criterion commonly used in nursing practice assessments, in which ≥ 80% of the maximum possible score indicates good performance [16]. Accordingly, mean scores ≥ 4.0 (80%) were classified as “good” practice, and lower scores as “poor” practice.

The third section ranked nine barriers (1 = *most important*, 9 = *least important*) using the HTF National Clinical Alarms Survey 2011 tool, which was used in a recent survey and demonstrated acceptable reliability, with a coefficient analysis of 0.71 [6].

#### 2.3.2. Embedded Qualitative Questions

To enrich the quantitative data and capture contextual insights, two open‐ended questions were included at the end of the questionnaire:

1. In your own words, what are the main challenges or barriers that make it difficult for you to respond to clinical alarms promptly and effectively?
2. Please describe any situation or condition that you think contributes most to alarm fatigue or delayed alarm response in your unit.

To ensure clarity, participants were instructed that these questions referred to their own direct experiences rather than observations about colleagues.

### 2.4. Validity and Reliability

#### 2.4.1. Quantitative Instrument

The questionnaire’s content validity was evaluated using the content validity assessment, reviewed by three specialized research professors in critical care nursing. The comments led to some revision to optimize clarity and relevance of questions. The pilot study involving 15 CCNs assessed clarity, understandability, and ease of administration and resulted in reorganization of tables to improve presentation. The pilot participants were eliminated from the study to prevent bias. The internal consistency reliability of the 9‐item practice scale was assessed using Cronbach’s alpha in the final sample (*n* = 146), yielding *α* = 0.78, which indicates acceptable internal consistency.

Content validity for the open‐ended question was reviewed by three experts in critical care nursing to ensure clarity and relevance. Credibility was maintained through careful and repeated review of participants’ responses, iterative coding, and reflective analysis to ensure consistency and accuracy in theme generation.

### 2.5. Data Collection Process

Following ethical approval, the principal investigator met with head nurses in CCUs of selected hospitals during August 2025 to explain the study’s purpose and obtain lists of eligible nurses’ characteristics and experiences. A total of 175 questionnaires were distributed. Of these, 146 were completed and returned, yielding a response rate of 83.4%. Written informed consent was obtained, and participants completed a structured, self‐reported questionnaire. Each participant was assigned a code for tracking. The researcher was present while the questionnaire was being completed to avoid any loss of data and collected the questionnaire on a daily basis.

### 2.6. Data Analysis

#### 2.6.1. Quantitative Analysis

SPSS Version 28 was used to analyze data (*n* = 146). Descriptive statistics described participant characteristics and nursing practice scores. One statement (“I ignore alarms every time they beep”) was reverse‐coded. For continuous variables, Pearson and Spearman’s rank‐order correlations investigated participant characteristic and practice score associations as appropriate. For categorical variables, *t*‐tests and ANOVA tested participant characteristic and practice score associations. A multiple linear regression analysis determined independent predictors of practice (all demographic and professional variables). Regression assumptions (linearity and normality of residuals and homoscedasticity and multicollinearity of predictor variables) were checked, and statistical significance was established at *p* < 0.05. Explanatory power of the model was expressed using *R* ^2^ and adjusted *R* ^2^.

#### 2.6.2. Qualitative Analysis

Narrative responses to the two open‐ended questions were transcribed verbatim by the principal investigator and analyzed thematically using Braun and Clarke’s [17] six‐step framework [17]. Codes were generated inductively and grouped into categories through reflective analysis until the final themes were established.

After separate analyses, qualitative themes were compared with quantitative findings to identify areas of convergence or divergence. Integration was primarily performed at the interpretation stage, particularly in explaining alarm‐management barriers and predictors.

### 2.7. Ethical Consideration

Ethical approval was obtained from the Ethical Committee of Palestine Polytechnic University (Approval no. EA/2025/85). Written informed consent was obtained from all participants prior to their involvement in the study. Participant rapport was established, and individuals were informed about the voluntary nature of their participation. Privacy during data collection was ensured by conducting it in a comfortable, private setting. Additionally, participants were assured that all personal information would be protected, secured, and kept confidential. To protect participant identities, all collected data were anonymized by assigning unique codes to each participant, removing any personally identifiable information. The study adhered to the ethical standards outlined in the Declaration of Helsinki regarding the rights, safety, and well‐being of research participants.

## 3. Result

### 3.1. Quantitative Findings

A total of 146 CCNs participated in the study. The mean age was 29 years (SD = 5.30), with a median of 25 and an interquartile range (IQR) of 25–32. More than half were female (52.7%), and the majority were married (61.0%). 17.1% had a master’s degree or higher. 28.1% had more than 10 years of CCU experience. Less than half (47.9%) had received prior training in clinical alarm management (Table 1).

The findings show that 45.2% of the participants reported always assessing the cause of an alarm when it beeped, while 39.0% always checked and assessed the patient’s condition upon alarm activation. Conversely, only 10.9% reported never ignoring alarms every time they beep. 34.2% always ensured proper skin preparation before electrode placement (Table 2).

**Table 2:** Nurses’ practices in the management of clinical alarms.

Variable	Never *N* (%)	Rarely *N* (%)	Sometimes *N* (%)	Often *N* (%)	Always *N* (%)	*M* (SD)
I ensure proper skin preparation of patients before placing electrodes	8 (5.5)	20 (13.7)	43 (29.5)	25 (17.1)	50 (34.2)	3.36 (1.23)
I change the patients’ electrodes daily	7 (4.8)	24 (16.4)	53 (36.3)	25 (17.1)	37 (25.3)	3.41 (1.17)
I assess the cause of the alarm beep when it alarms.	4 (2.7)	11 (7.5)	31 (21.2)	34 (23.3)	66 (45.2)	4.00 (1.10)
I disable the alarm only after assessing the patient and addressing the cause each time it beeps.	11 (7.5)	13 (8.9)	44 (30.1)	44 (30.1)	34 (23.3)	3.52 (1.16)
I pause the alarm only while actively assessing the patient each time it beeps.	5 (3.4)	19 (13.1)	55 (37.7)	48 (32.9)	19 (13.0)	3.39 (0.98)
I reset the alarm limits every time alarms beep	11 (7.5)	36 (24.7)	45 (30.8)	28 (19.2)	26 (17.8)	3.15 (1.19)
I reset alarm settings of the machines each time I admit a new patient	13 (8.9)	15 (10.3)	34 (23.3)	40 (27.4)	44 (30.1)	3.59 (1.26)
I check and assess the patient’s condition every time the alarm beeps	3 (2.1)	15 (10.3)	30 (20.5)	41 (28.1)	57 (39.0)	3.91 (1.09)
^∗^I ignore alarms every time they beep	16 (10.9)	64 (43.8)	48 (32.9)	11 (7.5)	7 (4.8)	3.48 (0.95)

*Note: N*, number; *M*, mean.

Abbreviation: SD, standard deviation.

^∗^Reverse coded item.

A cutoff point of ≥ 80% (> 4.0) was applied based on Bloom’s criteria; 34.2% of nurses demonstrated good practice, and 65.8% demonstrated poor practice. The overall mean practice score was 3.56 out of 5, equivalent to an average performance level of 71%.

### 3.2. Barriers to Effective Alarm Management

The most highly ranked barriers were inadequate staffing to respond promptly to alarms (Rank 1), frequent false alarms leading to reduced attention (Rank 2), and difficulty in understanding alarm priority (Rank 3). The least frequently reported barriers were difficulty in hearing alarms (Rank 8) and difficulty in identifying the source of alarms (Rank 9) (Table 3).

**Table 3:** Barriers to effective management of clinical alarms.

Barriers	Mean^a^	Rank^b^
Inadequate staff to respond to alarms as they occur	3.37	1
Frequent false alarms, which lead to reduced attention or response	3.50	2
Difficulty in understanding the priority of an alarm	3.89	3
Noise competition from non‐clinical alarms and pages	4.23	4
Overreliance on alarms to call attention to patient problems	4.30	5
Lack of training on alarm systems	4.34	6
Difficulty in setting alarms properly	4.39	7
Difficulty in hearing alarms when they occur	4.50	8
Difficulty in identifying the source of an alarm	4.50	9

^a^Mean ranking for the item.

^b^Ranking of the means.

### 3.3. Qualitative Findings From Open‐Ended Questions

Analysis of responses to the two open‐ended questions produced four overarching themes consistent with the quantitative results:

1. Alarm burden and desensitization: Nurses reported being overwhelmed by frequent false alarms, describing feelings of frustration, distraction, and delayed response. “Sometimes there are too many false alarms, and we stop reacting quickly,” one nurse noted.
2. Staffing and workload challenges: Participants emphasized high patient‐to‐nurse ratios and excessive workload as key obstacles to timely alarm management. “We are too few nurses to manage both patients and alarms at once.”
3. Limited training and organizational support: Many nurses highlighted a lack of structured training, absence of formal policies, and reliance on informal learning.
4. Environmental and technical barriers: Noise, overlapping device sounds, and malfunctioning sensors hindered quick identification and response.

### 3.4. Predictors of Alarm Management Practice

Preliminary analyses were conducted to assess associations between participant characteristics and alarm‐management practice. Age showed a significant negative correlation with practice scores (*ρ* = −0.18, *p* = 0.027), and years of experience showed a weaker but significant negative correlation (*ρ* = −0.15, *p* = 0.049). Independent‐samples *t*‐tests indicated significantly higher practice scores among female nurses (*t* = 4.18, *p* < 0.001) and among nurses with prior alarm training (*t* = −3.15, *p* ≤ 0.05). One‐way ANOVA revealed no significant differences across marital status or education level.

Variables examined in the bivariate analyses were then entered into a multiple linear regression model to identify independent predictors of alarm‐management practice (*R* = 0.494, *R* ^2^ = 0.244, Adjusted *R* ^2^ = 0.212). The regression highlighted three key predictors. Female nurses demonstrated stronger alarm management practices (*β* = 0.31, *p* < 0.001). Younger age (*β* = −0.21, *p* = 0.025), and prior training (*β* = 0.22, *p* = 0.004) were significant independent predictors (Table 4).

**Table 4:** Predictors of alarm management practice (*N* = 146).

Predictors	*B*	Std. error	*β*	*t*	*p* value
Age	−0.02	0.013	−0.21	−2.24	0.025
Gender	0.49	0.10	0.316	3.99	0.000
Marital status	−0.01	0.11	−0.008	−0.09	0.882
Level of education	0.13	0.09	0.113	1.44	0.379
Experience	−0.05	0.07	−0.068	−0.66	0.443
Alarm training	0.29	0.10	0.228	2.92	0.004

*Note: B*, unstandardized coefficients; *β*, standardized regression coefficient; SE, standard error; *t*, *t* value.

## 4. Discussion

### 4.1. Nurses’ Practices in the Management of Clinical Alarms

This study assessed CCNs’ clinical alarm practices, their predictors, and barriers in government hospitals of the Southern West Bank of Palestine. Quantitative findings showed that the majority of participating nurses scored below the expected level of alarm management practices. The best practices were checking alarm causes and checking patients’ status, and the poorest was resetting alarm limits. The qualitative findings also mirrored this, as nurses discussed focusing on priority alarms but disregarding habitual nuisance alarms. While the ≥ 80% cutoff is widely used in studies employing Bloom’s criteria, it represents a pragmatic rather than aspirational threshold. Meng’anyi et al. [4] described alarm‐management performance as ideally reaching 90%–100%, which suggests a higher expected standard. Therefore, using an 80% cutoff may provide a conservative estimate and may underestimate the true gap between actual and optimal practice. The results are consistent with previous Palestinian surveys in the central and northern West Bank, in which ICU nurses with 60% had moderate‐to‐high levels of alarm fatigue, strongly correlated with stress [3]. This study adds to that evidence by presenting real‐world implementation and demonstrating how alarm burden is expressed in terms of selective response.

Regionally, Egyptian [13] and Iranian [12] studies also mentioned poor practice, habitual disabling of alarms, and gaps in education. Lebanese evidence also showed desensitization of nurses and low alarm customization [14]. At an international level, distracted attention by false alarms was mentioned by South Korean [10] and Taiwanese [7] nurses, in alignment with our qualitative findings of prioritization strategies. However, U.S. surveys reported improved practice through formal education [8], pointing to the potential in Palestine for organizational interventions.

### 4.2. Barriers to Clinical Alarm Management

Top‐ranked barriers were inadequate staff, frequent false alarms, and difficulty understanding alarm priority. Qualitative findings reinforced these results: Nurses described short staffing and overwork as reasons for late response. “Short staffing results in delayed responding to non‐urgent alarms, compromising patient safety.” These barriers were consistent with Palestinian evidence by Salameh et al. [3], in which stress correlated strongly with alarm fatigue and, by implication, concluded that environmental factors and workload adversely affect safe practices. Similar systemic challenges in staffing, workload, and resource constraints have been reported in Palestinian emergency and critical care settings [18–21], indicating that alarm‐management difficulties are part of a broader pattern of resource‐driven limitations in Palestinian ICUs.

These findings were consistent with regional studies, whereas false alarms were cited in Iran [12]. A Lebanese survey listed nuisance alarms and staffing deficits [14]. However, an Egyptian study cited a lack of training as a major barrier [13], while in this study, lack of training was ranked as the sixth‐ranked barrier. The international literature corroborates these challenges. Frequent false alarms and low staffing levels decreased nurses’ responsiveness in Ireland [5]. Barriers in South Africa were challenges in alarm setting, insufficient training, and prioritization issues [6].

### 4.3. Predictors of Alarm Management Practice

Regression analysis identified female sex and pretraining as positive predictors and age as predicting poor practice. Qualitative results confirmed these tendencies: Many nurses reported “learning through experience” as a result of the lack of formal programs. Ayed et al. [22] similarly emphasized that poor work environments and reduced professional quality of life contribute to stress and diminished clinical performance among Palestinian ICU nurses, reinforcing the significance of addressing alarm fatigue within broader occupational wellbeing frameworks. Regionally, training has repeatedly been highlighted as essential. In Egypt, inadequate training correlated with unsatisfactory practice [13], and in Iran and Lebanon, nurses requested more structured education [12, 14]. Internationally, the connection between training and enhanced practice is strong. American and European investigations indicate enhanced alarm response through structured, repeated training [8, 11].

The study finding that female nurses practiced better could reflect contextual variations. Although gender emerged as a significant predictor in the regression model, the study did not indicate that female nurses in the southern West Bank have greater access to alarm‐related training. The training rates were similar between the genders, although the relationship between gender and improved practices was still significant after adjustment for training. This finding indicates that it might be related more to the role played in the context rather than the availability of training, as indicated by the differences seen in alarm management practices between the gender groups. Further research can explore the impact of cultural issues associated with the gender role in influencing the adoption of practices related to alarm management in the CCUs.

The negative relationship between the older participants and the study variables raises interesting implications. This might be because the younger nurses adapted well to the advancements in alarm technology, as there was also a relationship between the variables in the Lebanon study, where it was related to the perception of smart alarms [14]. The improved accuracy in the younger nurses might also be attributed to training updates, as the young Palestinian nurses hold more progressive views concerning the role of the profession as well as responsibilities [23].

The final regression model explained 24.4% of the variance in alarm management practice scores (adjusted *R* ^2^ = 0.212), indicating a modest but clinically meaningful fit and suggesting that other unmeasured factors (e.g., organizational culture, unit‐specific protocols, or individual coping styles) also influence practices.

The findings also underscore the role of organizational support, which was emphasized in participants’ qualitative narratives but underrepresented in prior Palestinian studies. Effective alarm management depends not only on individual competence but also on managerial policies that ensure adequate staffing ratios, access to device training, and maintenance of alarm equipment [11, 18, 24]. The absence of formal institutional frameworks for alarm response may explain inconsistencies in practice across CCUs.

## 5. Strengths and Limitations

This research is the initial inquiry into Palestine CCNs’ practices about clinical alarms as well as their barriers. Including the southern West Bank and the use of convenience sampling, while practical in this setting, limits the generalizability of the findings beyond the participating governmental hospitals. As a cross‐sectional study, cause‐and‐effect links cannot be determined. Moreover, as the study relied exclusively on self‐reported practices, social desirability bias and recall bias may have led participants to over‐report adherence to recommended alarm management behaviors. The coexistence of internal time indicators with an external frequency scale may introduce ambiguity. Coding single‐authored qualitative data might have augmented interpretation bias. Additionally, the qualitative data were collected via two brief written open‐ended questions rather than semi‐structured interviews, which restricted the depth and richness. Future studies with multisite samples of a large size and with several coders might increase transferability as well as robustness for results.

## 6. Implications and Recommendations

### 6.1. Clinical Practice

- Optimize alarm management by offering formal and regular training programs based on local needs. This recommendation reflects the predictive value of training identified in our regression analysis, even though lack of training was not the highest‐ranked barrier. Training demonstrated a meaningful influence on practice and was consistently echoed in qualitative feedback, and it represents the most feasible and controllable intervention in low‐resource settings.
- Enhance nurse‐to‐patient staffing ratios to lessen the workload barriers to timely response to alarms.
- Promote policies that support systematic individualization of alarm settings to reduce hassle alarms.

### 6.2. Education

- Add alarm management modules to nursing curricula and lifelong professional development education.
- Use simulation‐based education strategies to reduce the theory‐practice gap in clinical settings.

### 6.3. Policy and Organization

- Develop a hospital‐wide policy with alarm prioritization and response algorithms.
- Encourage investment in newer technologies for alarms that include filtering or intelligent systems to minimize false alarms.
- Engage leadership in addressing system‐level issues such as staffing ratios and resource limitations.

### 6.4. Research

- Conduct larger‐scale, multisite studies in Palestine to confirm these findings and examine the long‐term impact of the interventions.
- Investigate the influence of organizational culture on alarm management and professionalism in teamwork.

## 7. Conclusion

This study emphasizes that alarm management practice by CCNs in the southern West Bank is mostly below standard, with excessive false alarms, staffing workload shortages, and difficulties prioritizing alarms being the most significant barriers. Gender, age, and previous training predictors affected practice, whereas qualitative results emphasized workload, desensitization, and insufficient organizational support. These results indicate that alarm fatigue in Palestine reflects global issues but can be compounded by contextual constraints such as staffing shortages and limited resources. Moving forward, targeted staff training, strengthened educational programs, and clear policy directives on alarm safety should be prioritized to enhance nurses’ performance and reduce alarm‐related risks in critical care settings.

## Conflicts of Interest

The authors declare no conflicts of interest.

## Author Contributions

Fuad Farajalla: conceptualization, study design, data collection, qualitative analysis, drafting of the manuscript, and coordination of revisions. Mousa Farajallah: revised the manuscript through statistical analysis and interpretation of quantitative data and contributed to addressing the reviewers’ and editor’s major revision comments. Nesreen Alqaissi: contributed to the qualitative analysis and reviewed and edited the revised manuscript. Mohammad Qtait: contributed to addressing the reviewers’ and editor’s major revision comments.

## Funding

The author received no financial support for the research, authorship, and/or publication of this article.

## Supporting Information

Additional supporting information can be found online in the Supporting Information section.

The following supporting files are available with this article:

## Supporting information


**Supporting Information 1** Study Questionnaire: The structured questionnaire used in the study, including demographic information, alarm management practices, barriers, and open‐ended questions.

**Supporting Information 2** STROBE Checklist: A completed Strengthening the Reporting of Observational Studies in Epidemiology (STROBE) checklist to ensure transparent reporting of the quantitative component.

## Data Availability

The raw data underlying the findings of this study are provided as an Excel file submitted alongside the manuscript. The dataset is fully anonymized and contains no personally identifiable information.
