# Supplementary material for: Critical Care Nurses’ Practices in Clinical Alarm Management: Barriers and Predictors From a Mixed‐Methods Study in the Southern West Bank, Palestine
Source: Nurs Res Pract. 2026 Jan 11;2026:4564347. doi: 10.1155/nrp/4564347 (PMC12791022; doi:10.1155/nrp/4564347)
Supplement: Supplementary file 1 — Supporting Information 1 Study Questionnaire: The structured questionnaire used in the study, including demographic information, alarm management practices, barriers, and open‐ended questions. [file NRP-2026-4564347-s002.docx]

**Research Questionnaire**

**The research team is conducting a study on:**

**Barriers Affecting Clinical Alarm Practices and Their Determinants among Critical Care Nurses: A Mixed-Methods Study from the Southern West Bank.**

You have been selected as part of the study sample. We kindly request your assistance by answering the survey questions accurately and objectively as it has a significant impact on obtaining accurate results. Please note that all information will be kept strictly confidential and is for research purposes only. Participation in the study is voluntary, and therefore, there is no need to write your name or any information indicating your identity.

Additionally, the research team is prepared to provide you with the study results upon its completion if requested.

Research Team: Fuad W. Farajallah

Email: [Fuad.farajalla@gmail.com](mailto:Fuad.farajalla@gmail.com)

Phone : 0568537379

**We are grateful for your kind cooperation.**

**Section One: Sociodemographic Data**

- **Age:_______**
- **Gender:**

 Male

 Femal**e**

- **Marital status:**

 Single  Married  Other

- **Level of Education :**

**** Diploma  Bachelor's Degree  Master's Degree or Higher

- **Critical Care Units`s Experience:**

 1-3 years 4-6 years  > 6 years

- **Clinical Alarm - Related Training Courses
  Have you attended any specific courses on alarms and their devices?**

**No**

**Yes,** Specify Cardiac Physiological Monitod Ventilators

 CRRT Machine  Other/ Specify ...........

**Section Two: Nurses` Practices When Monitoring Clinical Alarms**

**Please answer on the following items regarding clinical alarm practice:**

| **Variables** | **Never** | **Rarely** | **Sometimes** | **Often** | **Always** |
| --- | --- | --- | --- | --- | --- |
| I ensure proper skin preparation of patients before placing electrodes |  |  |  |  |  |
| I change the patients’ electrodes daily |  |  |  |  |  |
| I assess the cause of the alarm beep when it alarms. |  |  |  |  |  |
| I disable the alarm **only after** assessing the patient and addressing the cause each time it beeps. |  |  |  |  |  |
| I pause the alarm **only while** actively assessing the patient each time it beeps. |  |  |  |  |  |
| I reset the alarm limits every time alarms beep |  |  |  |  |  |
| I reset alarm settings of the machines each time I admit a new patient |  |  |  |  |  |
| I check and assess the patient’s condition every time the alarm beeps |  |  |  |  |  |
| I ignore alarms every time they beep |  |  |  |  |  |

**Section Three: Barriers for Effective Clinical Alarm Management**

**Please rank the following nine issues that inhibit effective management of clinical alarms 1 (Most important) to 9 (Least important):**

| **1**  **Most important** |
| --- |
| **2** |
| **3** |
| **4** |
| **5** |
| **6** |
| **7** |
| **8** |
| **9**  **Least important** |

| **Issues** | **Rank From  (1-9)** |
| --- | --- |
| **1.Difficulty** in setting alarms properly |  |
| **2.Difficulty** in hearing alarms when they occur |  |
| **3.Difficulty** in identifying the source of an alarm |  |
| **4.Difficulty** in understanding the priority of an alarm |  |
| **5.Frequent false alarms**, which lead to reduced attention or response to alarms when they occur |  |
| **6.Inadequate staff** to respond to alarms as they occur |  |
| **7.Over reliance** on alarm to call attention to patient problems |  |
| **8.Noise competition** from non-clinical alarms and pages |  |
| **9.Lack of training** on alarm systems |  |

**Open Question:**

**1. In your own words, what are the main challenges or barriers that make it difficult for you to respond to clinical alarms promptly and effectively?**

**2. Please describe any situation or condition that you think contributes most to alarm fatigue or delayed alarm response in your unit .**
