# Supplementary material for: Critical Care Nurses’ Practices in Clinical Alarm Management: Barriers and Predictors From a Mixed‐Methods Study in the Southern West Bank, Palestine
Source: Nurs Res Pract. 2026 Jan 11;2026:4564347. doi: 10.1155/nrp/4564347 (PMC12791022; doi:10.1155/nrp/4564347)
Supplement: Supplementary file 2 — Supporting Information 2 STROBE Checklist: A completed Strengthening the Reporting of Observational Studies in Epidemiology (STROBE) checklist to ensure transparent reporting of the quantitative component. [file NRP-2026-4564347-s001.docx]

**STROBE Checklist**

**Study Title:** Critical Care Nurses’ Practices in Clinical Alarm Management: Barriers and Predictors from a Mixed-Methods Study in the Southern West Bank, Palestine

| **Item No.** | **Recommendation** | **Reported on Page #** | **Notes** |
| --- | --- | --- | --- |
| **1(a)** | Indicate the study’s design with a commonly used term in the title or the abstract | Abstract | Mixed-methods, cross-sectional quantitative + qualitative open-ended questions |
| **1(b)** | Provide in the abstract an informative and balanced summary | Abstract | Background, aim, methods, sample, results, and conclusions summarized |
| **2** | Explain scientific background and rationale | Introduction p. 2–4 | Global, regional, and local context of alarm fatigue provided |
| **3** | State specific objectives | Introduction p. 4 | Aim to assess CCN alarm practices, barriers, and predictors |
| **4** | Present key elements of study design early in paper | Methods p. 5 | Cross-sectional, descriptive correlational design with embedded qualitative component |
| **5** | Describe setting, locations, dates | Methods p. 5 | Governmental hospitals, Southern West Bank, August 2025 |
| **6(a)** | Give eligibility criteria and participant selection | Methods p. 6 | Inclusion: CCNs currently employed, available, and consenting; Exclusion: nurse managers, <1 year experience |
| **7** | Define outcomes, exposures, predictors | Methods p. 7 | Outcome: practice score; predictors: age, gender, marital status, education, experience, training |
| **8** | For each variable, give sources of data and measurement | Methods p. 7 | Structured questionnaire (demographics, 9-item practice tool, barrier rankings); open-ended questions for qualitative data |
| **9** | Describe efforts to address potential bias | Methods p. 7 | Pilot study, expert review, reflective notes, anonymization, researcher presence during data collection |
| **10** | Explain how study size was arrived at | Methods p. 6 | Raosoft sample size calculator; n=132 minimum; 146 completed |
| **11** | Explain handling of quantitative variables | Methods p. 7 | Likert scale (5=always, 1=never); practice scores converted to percentages (>80% good, ≤80% poor) |
| **12(a)** | Describe statistical methods, including confounding control | Methods p. 8 | Descriptive statistics, Pearson correlations, t-tests, ANOVA, multiple linear regression |
| **12(b)** | Methods for subgroups/interactions | N/A | Not conducted |
| **12(c)** | Explain how missing data were addressed | Methods p. 6 | 146/175 completed (83.4%); minimal missing data, researcher presence minimized loss |
| **12(d)** | Analytical methods considering sampling strategy | Methods p. 6 | Convenience sampling acknowledged |
| **12(e)** | Describe any sensitivity analyses | N/A | None conducted |
| **13(a)** | Report numbers of individuals at each stage | Results p. 9 | 175 distributed, 146 analyzed |
| **13(b)** | Give reasons for non-participation | Results p. 9 | 29 not returned; reasons not detailed |
| **13(c)** | Consider use of a flow diagram | N/A | Not included |
| **14(a)** | Give characteristics of study participants | Results Table 1 p. 9 | Age, gender, marital status, education, experience, training |
| **14(b)** | Indicate number with missing data | Results p. 9 | Minimal missing data; assumed complete dataset |
| **15** | Report outcome events or summary measures | Results Tables 2–4 p. 9–10 | Practice scores, barrier rankings, regression outcomes |
| **16(a)** | Give unadjusted and adjusted estimates | Results Table 4 p. 10 | Regression coefficients, standardized coefficients, p-values |
| **16(b)** | Report category boundaries when continuous variables categorized | Methods p. 7 | Practice scores >80% good, ≤80% poor |
| **16(c)** | Translate estimates into absolute risk | N/A | Not applicable for regression |
| **17** | Report other analyses | Results p. 10 | Qualitative thematic analysis of open-ended responses |
| **18** | Summarize key results with reference to objectives | Discussion p. 11–12 | Practice rates, barriers, predictors aligned with study aims |
| **19** | Discuss limitations | Discussion p. 13 | Single-site, cross-sectional, self-report bias, single-author coding |
| **20** | Overall interpretation | Discussion p. 11–13 | Findings contextualized with global, regional, and local studies |
| **21** | Discuss generalisability | Discussion p. 13 | Limited to Southern West Bank CCNs due to single-site study |
| **22** | Source of funding and role of funders | Acknowledgments p. 14 | No funding reported |
